# Fault-Tolerant Control of ANPC Three-Level Inverter Based on Order-Reduction Optimal Control Strategy under Multi-Device Open-Circuit Fault

**DOI:** 10.1038/s41598-017-15000-9

**Published:** 2017-10-31

**Authors:** Shi-Zhou Xu, Chun-Jie Wang, Fang-Li Lin, Shi-Xiang Li

**Affiliations:** College of Electronic and Electrical Engineering, Henan Normal University, Xinxiang, 453007 China

## Abstract

The multi-device open-circuit fault is a common fault of ANPC (Active Neutral-Point Clamped) three-level inverter and effect the operation stability of the whole system. To improve the operation stability, this paper summarized the main solutions currently firstly and analyzed all the possible states of multi-device open-circuit fault. Secondly, an order-reduction optimal control strategy was proposed under multi-device open-circuit fault to realize fault-tolerant control based on the topology and control requirement of ANPC three-level inverter and operation stability. This control strategy can solve the faults with different operation states, and can works in order-reduction state under specific open-circuit faults with specific combined devices, which sacrifices the control quality to obtain the stability priority control. Finally, the simulation and experiment proved the effectiveness of the proposed strategy.

## Introduction

The ANPC three-level topology has the advantages of NPC three-level topology. What’s more, it can overcome the unbalanced power-loss generation of power devices caused by different switching frequency using selecting different zero switching states. It has a much higher system freedom and much flexible control strategies, which can use different PWM control strategies to control the system to realize the corresponding performances. Therefore, the ANPC inverter has been paid much attention by researchers all over the world, and becomes mature as a system, but at present, the multi-device open-circuit fault is one of the urgent faults that need to be solved.

Right now, the fault-tolerant control strategy research on two-level inverter has been relatively mature and had many corresponding results. The most widespread topological structures of two-level fault-tolerant control are double winding redundant topology, bridge-arm redundant topology, switch-redundancy topology, three-phase four-bridge-arm fault tolerant topology, four-switch two-phase fault tolerant topology, three-phase H bridge topology, modular redundant topology, etc. The most frequently used control methods of two-level inverter fault-tolerant control are vector control, direct torque control, weak magnetic speed-up control and so on.

**Figure 1:**
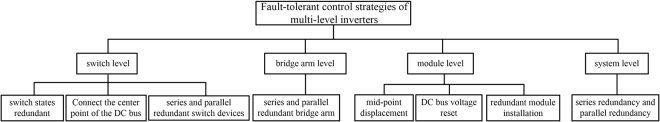
Categories of fault-tolerant control methods for multi-level inverters.

The multi-level inverter topologies are much more novel and complicated, so the fault-tolerant control research on them are much later. While, in recent years, multi-level inverters have been used widely in industrial field because of their inherent advantages, more output levels, less harmonic content, lower bus voltage acting on power devices, and so on, compared with two-level inverters. Therefore, the researchers in this field all over the world have paid much attention to multi-level inverters, and some achievements on fault-tolerant control of NPC(Neutral-Point-Clamped) three-level inverter have been made. The fault-tolerant control methods can be divided into four types, namely, switch level fault-tolerant control, bridge arm level fault-tolerant control, module level fault-tolerant control and system level fault-tolerant control, which are shown in Fig. 1.

The fault-tolerant control strategies of switch level can be divided into several kinds, and the most important three kinds are using the inherent redundant switch states^1–4^, connecting the center point of DC bus^5–12^, series and parallel redundant switch devices^13–16^. The strategies proposed in^17–21^ are the ones belong to bridge-arm level, which have a redundant bridge arm in parallel. When the bridge arm is open or short circuit during operating, the redundant bridge arm will replace it and keep the system working as normal. This redundant bridge-arm fault tolerant control costs too much and has a much less application. The topologies of the cascaded multilevel converter (CMC) and modular multi-level converter (MMC) have the feature of modularity, which can use the module-level fault-tolerant control methods. The module-level fault-tolerant control methods can be divided into three types, mid-point displacement, DC bus voltage reset, and redundant module installation. The system-level fault-tolerant control is the one install a redundant inverter to replace the fault one during the malfunction occurring, ensuring the performance of the whole system unchanged. The common system redundancy includes series redundancy and parallel redundancy. A comparison of fault-tolerant control strategies were shown in ref.^22^. A SBPM-based SOH monitor was proposed in ref.^23^, which provides a practical method for the realization of fault-tolerant control. An *in-situ* voltage fault diagnosis method based on the modified Shannon entropy was proposed in ref.^24^, which is capable of predicting the voltage fault in time through monitoring battery voltage during vehicular operations. Another practical method of design, control and analysis of fault tolerant soft-switching DC-DC converter was proposed in ref.^25^. Similarly, a variable slope trapezoidal reference signal based control for DC fault tolerant hybrid modular multilevel converter was studied in ref.^26^, which offers a very useful method for reference. In ref.^27^, the state-of-art equivalent circuit models for ultracapacitors are studied and the hybrid pulse power characterization test is conducted to collect the data for parameter identification, on which base the genetic algorithm is employed to extract the optimal model parameters to evaluate the model accuracy, complexity and robustness. In ref.^28^, a novel fractional-order model composed of a series resistor, a constant-phase-element (CPE), and a Walburg-like element, were proposed to emulate the UC dynamics. All these methods provided useful references for fault-tolerant control strategies.

The topology of active neutral-point clamped three-level inverter became a research hot spot on the moment it was proposed. It can balance the power losses of inverter power devices by choosing four zero switching states to reduce the failure possibility of those devices with high switching frequency. So, the ANPC inverter has a much higher operation stability compared with NPC inverters. However, it is not enough and it is necessary to realize the fault-tolerant control for ANPC inverters to meet the high stability requirement of inverters used in some import special occasions. The design of hardware fault tolerant control architecture for wind energy conversion system with DFIG based on reliability analysis was presented in ref.^29^. The power loss and the single device failure of ANPC three-level inverter were analyzed in ref.^30^. The stabilities of NPC three-level inverter and ANPC three-level inverter were analyzed and compared in ref.^31^, which pointed out that the ANPC inverter has a much higher stability than NPC inverter when some power devices break down. The possible open-circuit and short-circuit faults of single device in ANPC inverter were analyzed in theory in ref.^32^, and the corresponding fault-tolerant control method was proposed. The simulation and experiment results proved the effectiveness of the proposed method. Meanwhile, The open-circuit and short-circuit faults of multiple devices in ANPC inverter were analyzed in theory, too, but without simulation and experiment. In this paper, on the basis of the existing researches, the fault-tolerant control strategy of ANPC three-level inverter will be studied further to improve the stability.

## Principle of Order-Reduction Optimal Control Strategy

**Figure 2:**
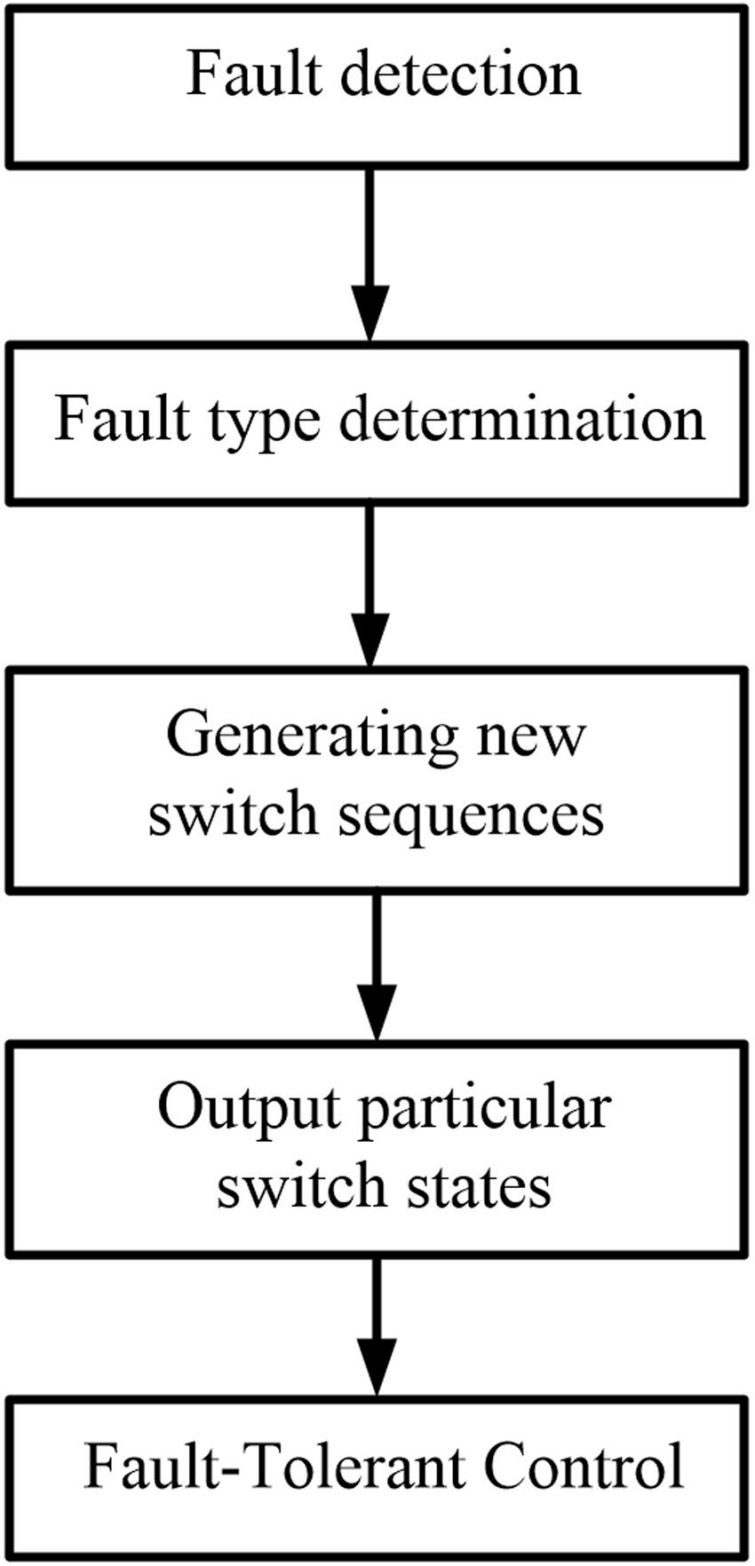
Flow chart of order-reduction optimal control strategy.

The flow chart of the order-reduction optimal control strategy is shown in Fig. 2.

**Figure 3:**
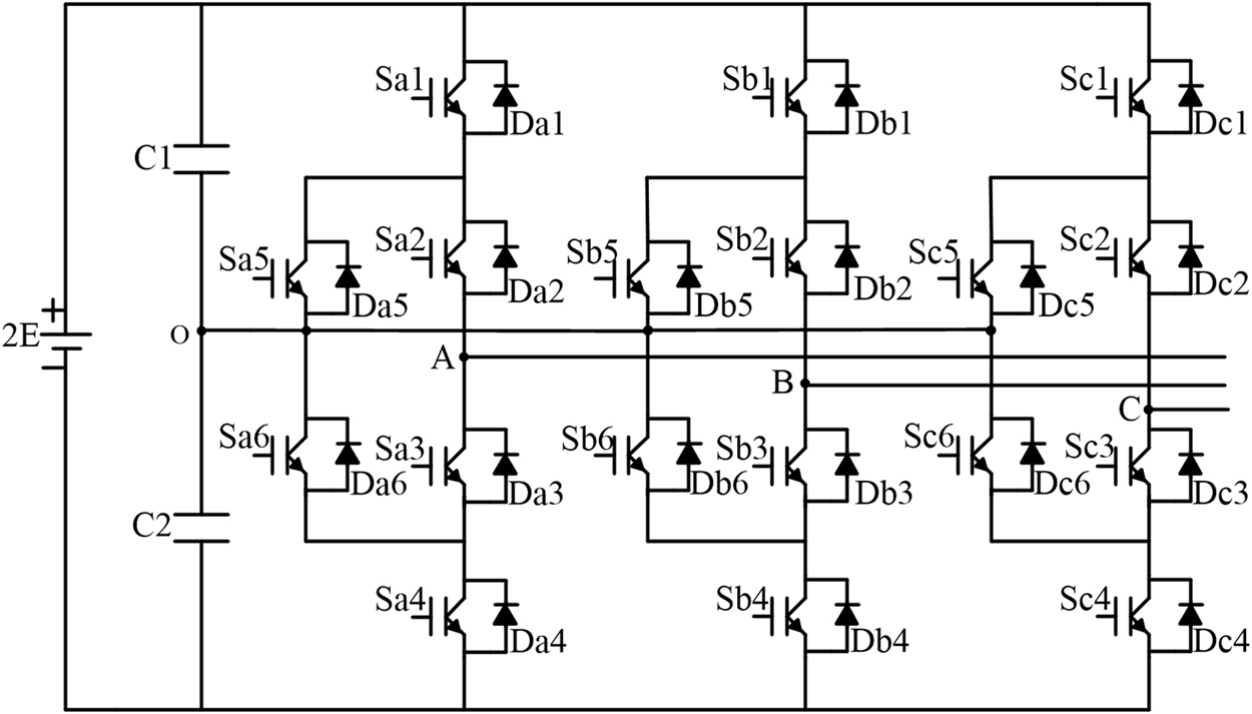
Topology of ANPC three-level inverter.

The topology of ANPC three-level inverter was shown in Fig. 3. It can be seen from the topology that the ANPC three-level inverter turn the two clamped diodes of each bridge arm from NPC three-level inverter into IGBT modules to add the flowing paths of the zero state.

The impacts caused by multiple devices open circuit of ANPC three-level inverter on the fault phases were summarized in Table 1. In Table 1, “healthy” means this phase works normally without fault device; “NRF” (No Reduction Fault) means this phase has faults, but this fault phase still can output three kinds of levels, “+”, “0”, and “−”; “NRF-2L” means has faults, but this fault phase only outputs two kinds of levels, “+” and “−”; “RF” (Reduction fault) means has faults, and this fault phase only outputs “0” level. For example, if Sa1/Da1, Sa2/Da2, Sa3/Da3 and Sa4/Da4 are healthy, even though both Sa5 and Sa6 fail at the same, this fault phase will still work in “NRF” state, and output “+”, “0”, and “−”; if Sa2/Da2 and Sa5/Da5 works normally, even though all the power devices of this fault phase are in open-circuit state, this fault phase still works under “RF” state, and output “0” level.

**Table 1:** Output states of fail phases under multiple devices open-circuit failure in single phase.

Sa1/Da1 Sa4/Da4	Sa2	Da2	Sa3	Da3	Sa5	Da5	Sa6	Da6	Phase state
Ok	Ok	Ok	Ok	Ok	Fail	Ok	Fail	Ok	NRF
Ok	Ok	Ok	Ok	Ok	Ok	Fail	Ok	Fail	NRF
Ok	Ok	Ok	Ok	Ok	Ok	Ok	*Fail*	Fail	NRF
Ok	Ok	Ok	Ok	Ok	Fail	Fail	Ok	Ok	NRF
Ok	Ok	Ok	Ok	Ok	Fail	Fail	Fail	Fail	NRF-2L
Fail	Ok	Fail	Ok	Fail	Fail	Ok	Fail	Ok	RF
Fail	Fail	Ok	Fail	Ok	Ok	Fail	Ok	Fail	RF
Fail	Ok	Ok	Fail	Fail	Ok	Ok	Fail	Fail	RF
Fail	Fail	Fail	Ok	Ok	Fail	Fail	Ok	Ok	RF

**Figure 4:**
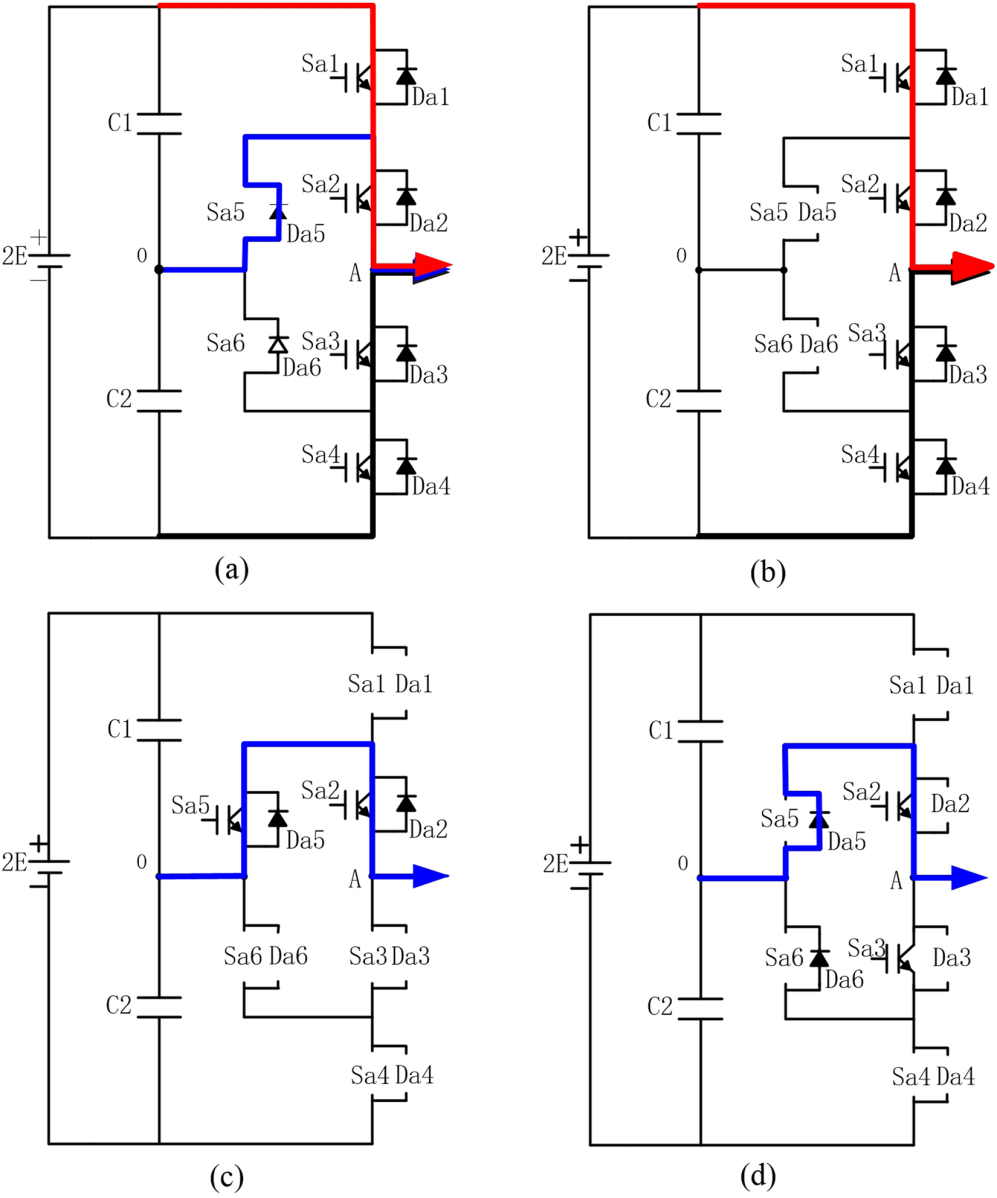
Current flow paths under multi-device open-circuit faults in 3-L ANPC inverter. (**a**) Sa5 and Sa6 in open-circuit state; (**b**) Sa5/Da5 and Sa6/Da6 in open-circuit state; (**c**) Sa1/Da1, Sa3/Da3, Sa4/Da4 and Sa6/Da6 in open circuit state; (**d**) Sa1/Da1, Da2, Da3, Sa4/Da4, Sa5 and Sa6 in open-circuit state.

It can be seen from Table 1 that when Sa1/Da1, Sa2/Da2, Sa3/Da3 and Sa4/Da4 are all work in normal state, even if Sa5 and Sa6 work in open-circuit fault state, this fault phase are still under “NRF” state, and output “+”, “0” and “−” levels, as shown in Fig. 4(a); when Sa1/Da1, Sa2/Da2, Sa3/Da3 and Sa4/Da4 are all work in normal state, and Sa5/Da5 and Sa6/Da6 work in open-circuit fault state, this fault phase works in “NRF-2L” state, outputs “+” and “−” levels, as shown in Fig. 4(b); when Sa2/Da2 and Sa5/Da5 work in normal state, even if all the power devices in this fault phase are in open-circuit fault state, it still stays in “RF” state, and outputs “0” level, as shown in Fig. 4(c); when Sa2, Sa3, Da5 and Da6 work in normal state, even if all the other devices in this fault phase are in open-circuit fault, this fault phase still works under “RF” state, and only outputs “0” level, as shown in Fig. 4(d).

According to the output states of each phase in Table 1, the fault-tolerant control method under multi-device open-circuit fault of three-phase ANPC inverter can be summarized, and is shown in Table 2.

**Table 2:** Operation states under fault-tolerant control with multi-device open-circuit fault.

	Phase A state	Phase B state	Phase C state
Mode 1	Healthy/NRF	Healthy/NRF	Healthy/NRF
Mode 2	NRF-2L	Healthy/NRF/NRF-2L	Healthy/NRF/NRF-2L
Mode 3	RF	Healthy/NRF/NRF-2L	Healthy/NRF/NRF-2L

**Figure 5:**
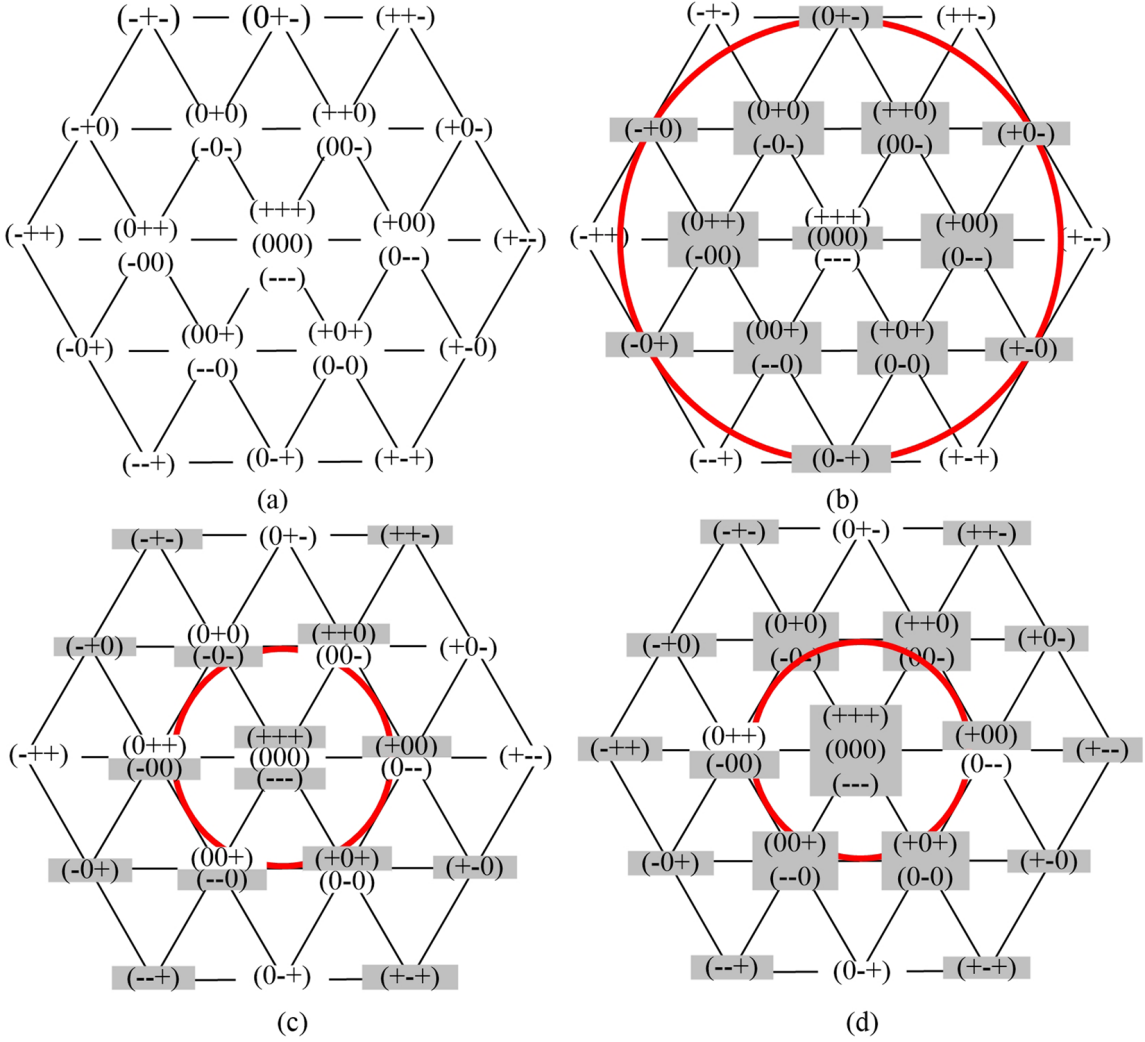
Voltage vector diagram of 3L-ANPC inverter under multi-device open-circuit fault.

When one phase of three-phase ANPC three-level inverter breaks down, the operation under fault-tolerant control can be divided into three modes according to the output states of fault phase. In mode 1 and 2, the maximum modulation is the same as normal operation, while in mode 3, its maximum modulation decreases to 0.577. What’s more, only under mode 1, the inverter outputs the same waveform quality as the normal one. The voltage vector diagram was introduced to illustrate the fault-tolerant control operation modes, and it is shown in Fig. 5. In Fig. 5, gray indicates the states that cannot output normally, and white indicates the states that can output normally.

When ANPC three-level inverter works under “healthy” or “NRF” state, each phase can still output “+”, “0” and “−”. The voltage vector diagram is shown in Fig. 5(a), and under this state, all the voltage vectors can still be used as normal to maintain the inverter work as normal. If one of the three phases works under NRF-2L state, the working condition of the inverter can be supposed to be much worse. All the three phases were supposed to be under NRF-2L state, and the voltage vector diagram is shown in Fig. 5(b), where the external hexagon still has six correct voltage vectors to be used. Therefore, the ANPC three-level inverter works under the same condition as the two-level inverter’s. Although the waveform quality is reduced, its maximum modulation is the same as the one in normal state.

When one phase of the ANPC three-level inverter is under RF state and the other two phases are under healthy or NRF states, the voltage vector is shown in Fig. 5(c). It can be seen from Fig. 5(c) that the six voltage vectors of the inner hexagon is still valid as normal, which means the fault tolerant control is useful under this condition, but the maximum modulation is reduced to 0.577. Compared with the proposed fault states former, there is a much worse operation state, one phase of the ANPC three-level inverter is under FR state and the other two phases both work under NRF-2L state. Under this state, the four valid voltage vectors are shown in Fig. 5(d). When the three-phase ANPC three-level inverter operates under this mode, its equivalent circuit is the same as four-switched three-phase inverter, and can realize the fault-tolerant control operation with the maximum modulation reduced to 0.577.

**Figure 6:**
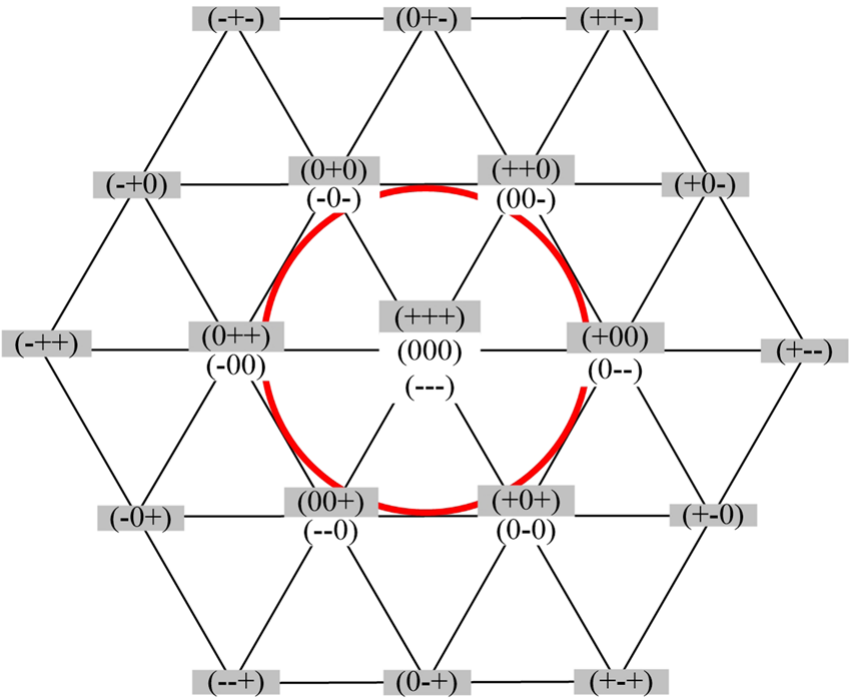
Voltage vector diagram of 3L-ANPC inverter using lower dc-link capacitor for fault tolerant operation.

When phase A and B both work under RF state, even though phase C works under healthy state, only (00−), (000) and (00+) are can be valid. Under this condition, the ANPC three-level inverter can not work with fault-tolerant control according to what has been analyzed before. However, when the topology of the inverter is recomposed and only one capacitor of the DC-bus side is used to generate a two-level voltage waveform, the fault-tolerant control can be realized. For example, if Sa1/Da1, Sb1/Db1 and Sc1/Dc1 goes into open-circuit state at the same time, the fault phases can only output “0” and “−” level, and the voltage vector diagram is shown in Fig. 6.

Under this condition, the fault-tolerant control mode of ANPC three-level inverter can be equalled to a two-level inverter, whose maximum modulation decreased to 0.577. In the same way, if Sa4/Da4, Sb4/Db4, and Sc4/Dc4 come into open-circuit fault at the same time, the fault phase can only output “+” and “0” and the whole system will still work with fault-tolerant control.

## Simulation and Experiment

### Simulation

The experiment parameters are: DC busbar voltage ${U}_{dc}=400V$, the upper and lower capacitance of DC side ${C}_{1}={C}_{2}=10mF$, the sampling frequency ${f}_{sw}=2KHz$, the modulation M = 0.8, frequency $f=50Hz$, the resistance load ${R}_{1}=8{\rm{\Omega }}\,$, $L=23mH$.

**Figure 7:**
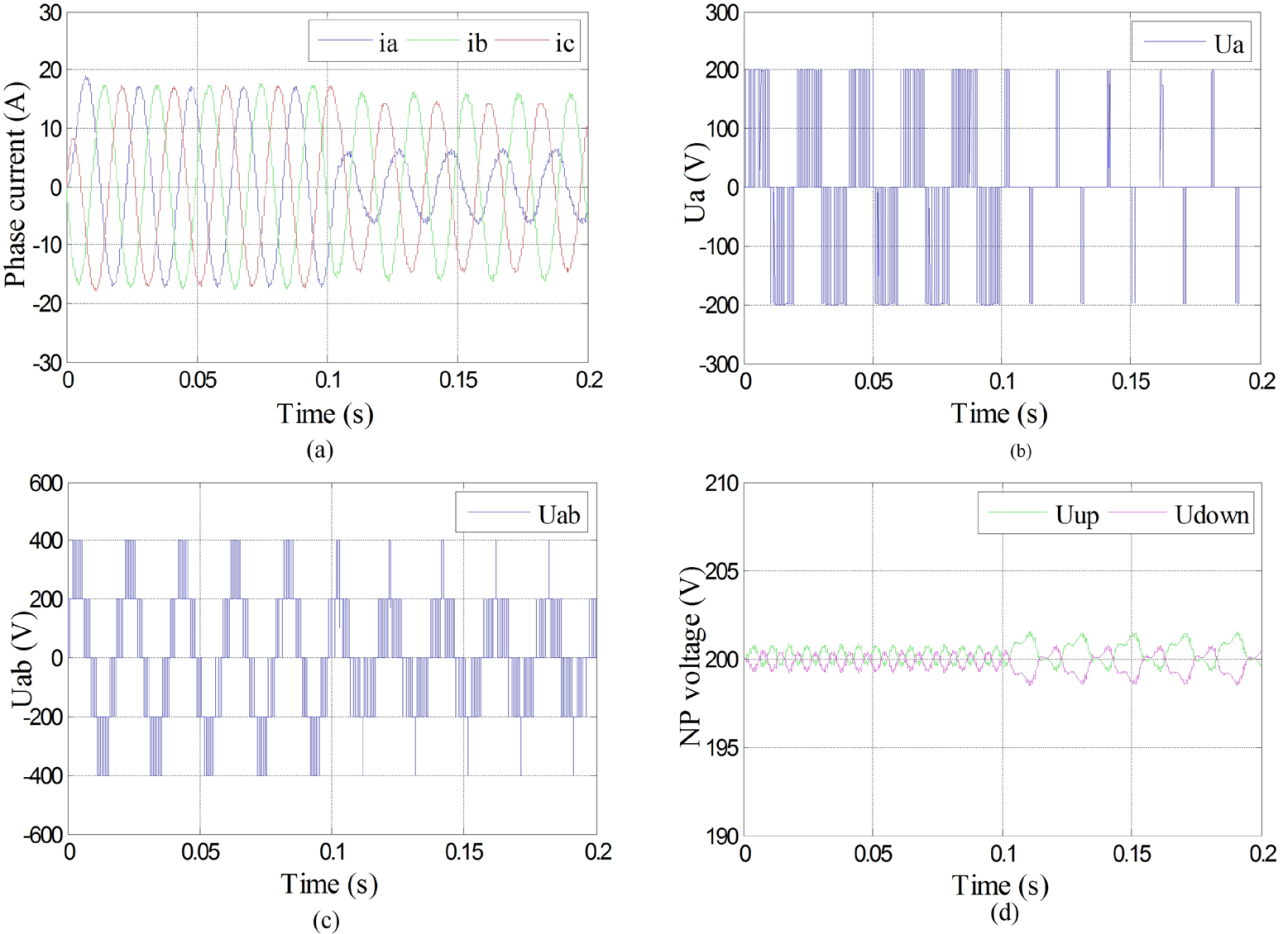
Phase current, phase voltage, line voltage and neutral-point voltage under Sa1 and Sa4 open-circuit fault. (**a**) Three-phase currents under Sa1 and Sa4 open-circuit fault; (**b**) Phase voltage of phase A under Sa1 and Sa4 open-circuit fault; (**c**) Line voltage between phase A and B under Sa1 and Sa4 open-circuit fault; (**d**) Neutral-point voltage under Sa1 and Sa4 open-circuit fault.

The waveforms of the output current of phase A, B and C, the phase voltage of phase A, the A-B line voltage, and the neutral-point voltage under open-circuit fault were shown in Fig. 7. It can be seen from the Fig. 7(a) that the three-phase currents are no longer symmetrical, and the current of phase A has been changed. The phase voltage of phase A and the line voltage of A-B, as shown in Fig. 7(b) and (c) respectively, have also been distorted when Sa1and Sa4 come into open circuit. Meanwhile, the neutral-point voltage, as shown in Fig. 7(d), has been shifted, but it still can keep balance.

**Figure 8:**
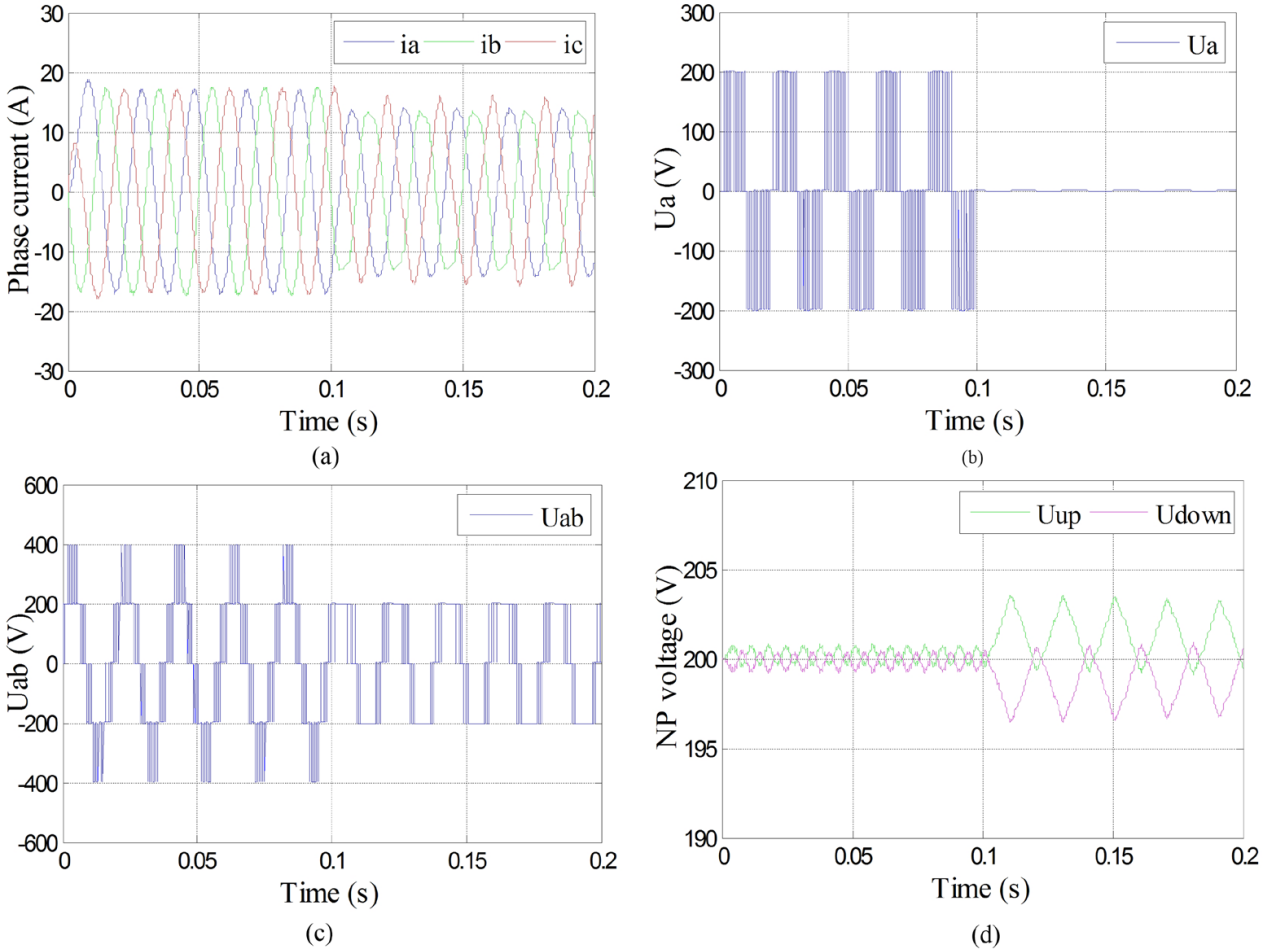
Phase current, phase voltage, line voltage and neutral-point voltage under Sa1 and Sa4 open-circuit fault with fault-tolerant control. (**a**) Three-phase currents under Sa1 and Sa4 open-circuit fault with fault-tolerant control; (**b**) Phase voltage of phase A under Sa1 and Sa4 open-circuit fault with fault-tolerant control; (**c**) Line voltage between phase A and B under Sa1 and Sa4 open-circuit fault with fault-tolerant control; (**d**) Neutral-point voltage under Sa1 and Sa4 open-circuit fault with fault-tolerant control.

The three-phase currents, phase voltage of phase A, line voltage between phase A and B, and the neutral-point voltage were shown in Fig. 7 under Sa1 and Sa4 open-circuit fault in phase A with fault-tolerant control. It can be seen from Fig. 8(a) that even though the amplitudes of three-phase currents decreased, they were maintained symmetry. It can be seen from Fig. 8(b) that the phase voltage of phase A changed into 0 with fault-tolerant control after the fault occurrence of phase A. It can be seen from Fig. 8(c) that even though the amplitude of line voltage between phase A and B decreased, they were maintained symmetry. It can be seen from Fig. 8(d) that the neutral-point voltage shifted a little under fault-tolerant control compared with normal condition, but it can still keep balance.

**Figure 9:**
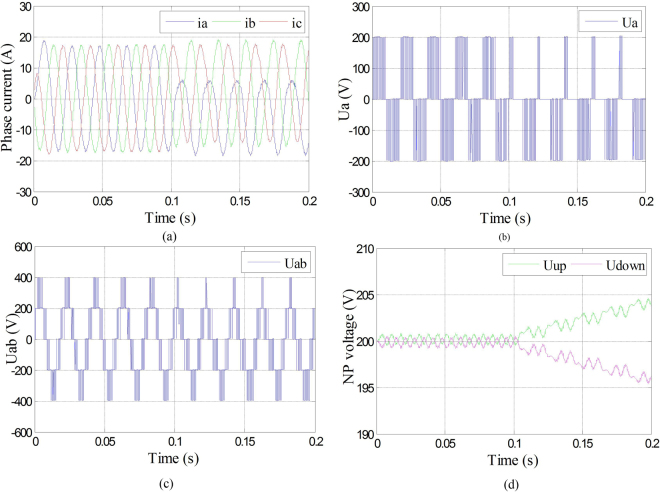
Phase current, phase voltage, line voltage and neutral-point voltage under Sa1 and Sa5 open-circuit fault. (**a**) Three-phase currents under Sa1 and Sa5 open-circuit fault; (**b**) Phase voltage of phase A under Sa1 and Sa5 open-circuit fault; (**c**) Line voltage between phase A and B under Sa1 and Sa5 open-circuit fault; (**d**) Neutral-point voltage under Sa1 and Sa5 open-circuit fault.

The three-phase currents, phase voltage of phase A, line voltage between phase A and B, and the neutral-point voltage were shown in Fig. 9 under Sa1 and Sa5 open-circuit fault in phase A. It can be seen from Fig. 9(a) that the phase current of phase A changed most significantly. It can be seen from Fig. 9(b–d) respectively that the phase voltage of phase A, line voltage between phase A and B, and the neutral-point voltage had also changed or shifted. Meanwhile, the balance of neutral-point voltage was lost.

**Figure 10:**
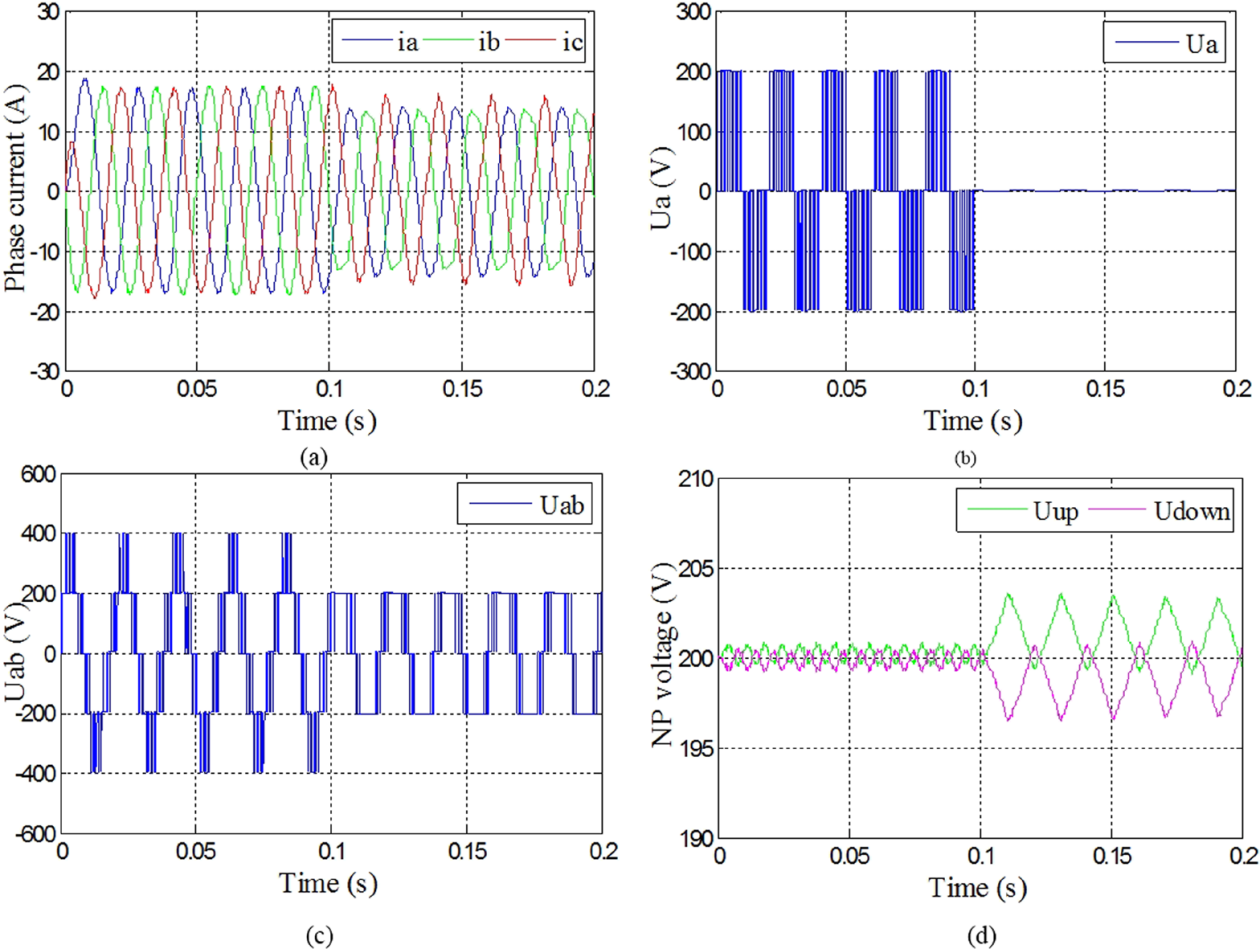
Phase current, phase voltage, line voltage and neutral-point voltage under Sa1 and Sa5 open-circuit fault with fault-tolerant control. (**a**) Three-phase currents under Sa1 and Sa5 open-circuit fault with fault-tolerant control; (**b**) Phase voltage of phase A under Sa1 and Sa5 open-circuit fault with fault-tolerant control; (**c**) Line voltage between phase A and B under Sa1 and Sa5 open-circuit fault with fault-tolerant control; (**d**) Neutral-point voltage under Sa1 and Sa5 open-circuit fault with fault-tolerant control.

The three-phase currents, phase voltage of phase A, line voltage between phase A and B, and the neutral-point voltage were shown in Fig. 10 under Sa1 and Sa5 open-circuit fault in phase A with fault-tolerant control. It can be seen from Fig. 10(a) that even though the amplitudes of three-phase currents decreased, they were maintained symmetry. It can be seen from Fig. 10(b) that the phase voltage of phase A changed into 0 with fault-tolerant control after the fault occurrence of phase A. It can be seen from Fig. 10(c) that even though the amplitude of line voltage between phase A and B decreased, they were maintained symmetry. It can be seen from Fig. 10(d) that the neutral-point voltage shifted a little under fault-tolerant control compared with normal condition, but it can still keep balance.

**Figure 11:**
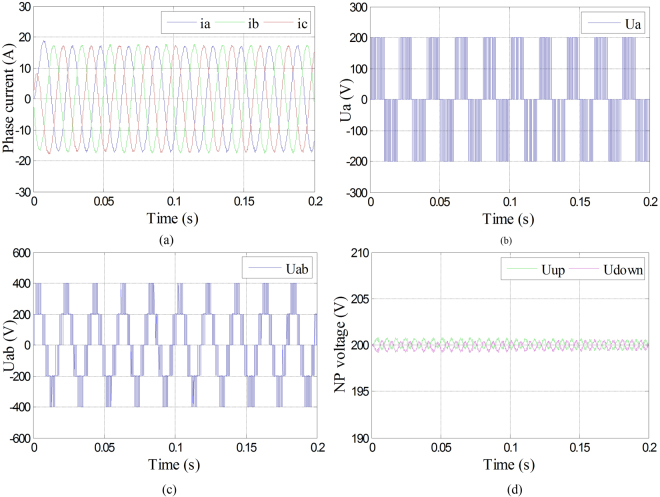
Phase current, phase voltage, line voltage and neutral-point voltage under Sa5 and Sa6 open-circuit fault with fault-tolerant control. (**a**) Three-phase currents under Sa5 and Sa6 open-circuit fault with fault-tolerant control; (**b**) Phase voltage of phase A under Sa5 and Sa6 open-circuit fault with fault-tolerant control; (**c**) Line voltage between phase A and B under Sa5 and Sa6 open-circuit fault with fault-tolerant control; (**d**) Neutral-point voltage under Sa5 and Sa6 open-circuit fault with fault-tolerant control.

The three-phase currents, phase voltage of phase A, line voltage between phase A and B, and the neutral-point voltage were shown in Fig. 11 under Sa5 and Sa6 open-circuit fault in phase A with fault-tolerant control. It can be seen from Fig. 11(a–d) respectively that the three-phase currents, phase voltage of phase A, line voltage between phase A and B decreased, neutral-point voltage were still kept as the same as the ones before fault occurrence. Therefore, when Sa5 goes into open-circuit fault, it can be maintained operation as normal without fault-tolerant control.

Since the symmetry of the ANPC topology, it is valid only to analyze the upper bridge arm of the fault phase during the multi-device open-circuit fault. What’s more, the open-circuit fault of diodes is the same as IGBT’s, so it will not be analyzed in detail repeatedly.

### Experiment

**Figure 12:**
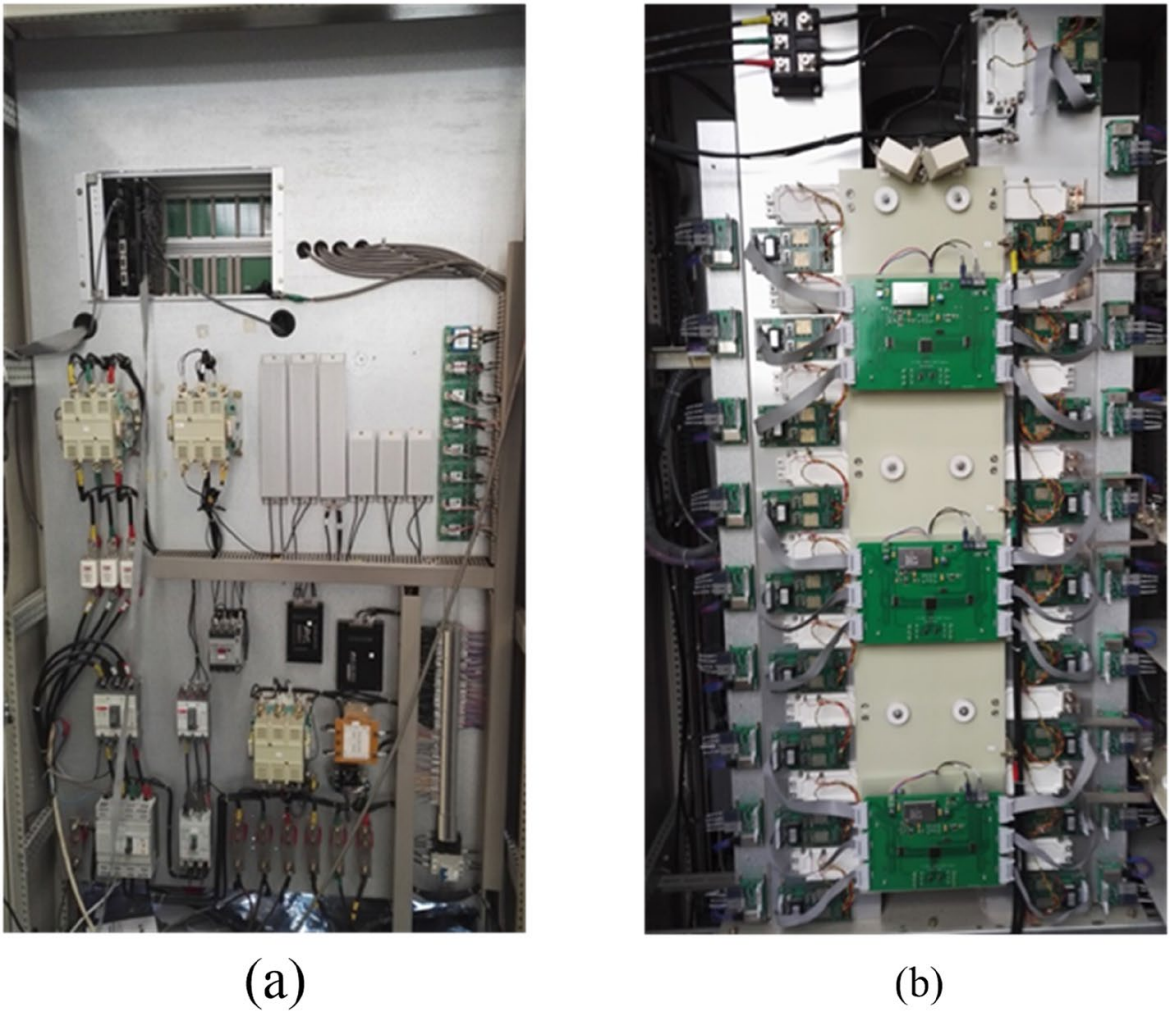
The experiment platform. (**a**) The front of the experiment platform; (**b**) The front of the experiment platform.

The experiment parameters are: DC busbar voltage ${U}_{dc}=400\,V$, the upper and lower capacitance of DC side ${C}_{1}={C}_{2}=10\,mF$, the sampling frequency ${f}_{sw}=2\,KHz$, the modulation M = 0.8, frequency $f=50\,Hz$, the resistance load ${R}_{1}=8\,{\rm{\Omega }}\,$, $L=23\,mH$. The experiment platform is shown in Fig. 12.

**Figure 13:**
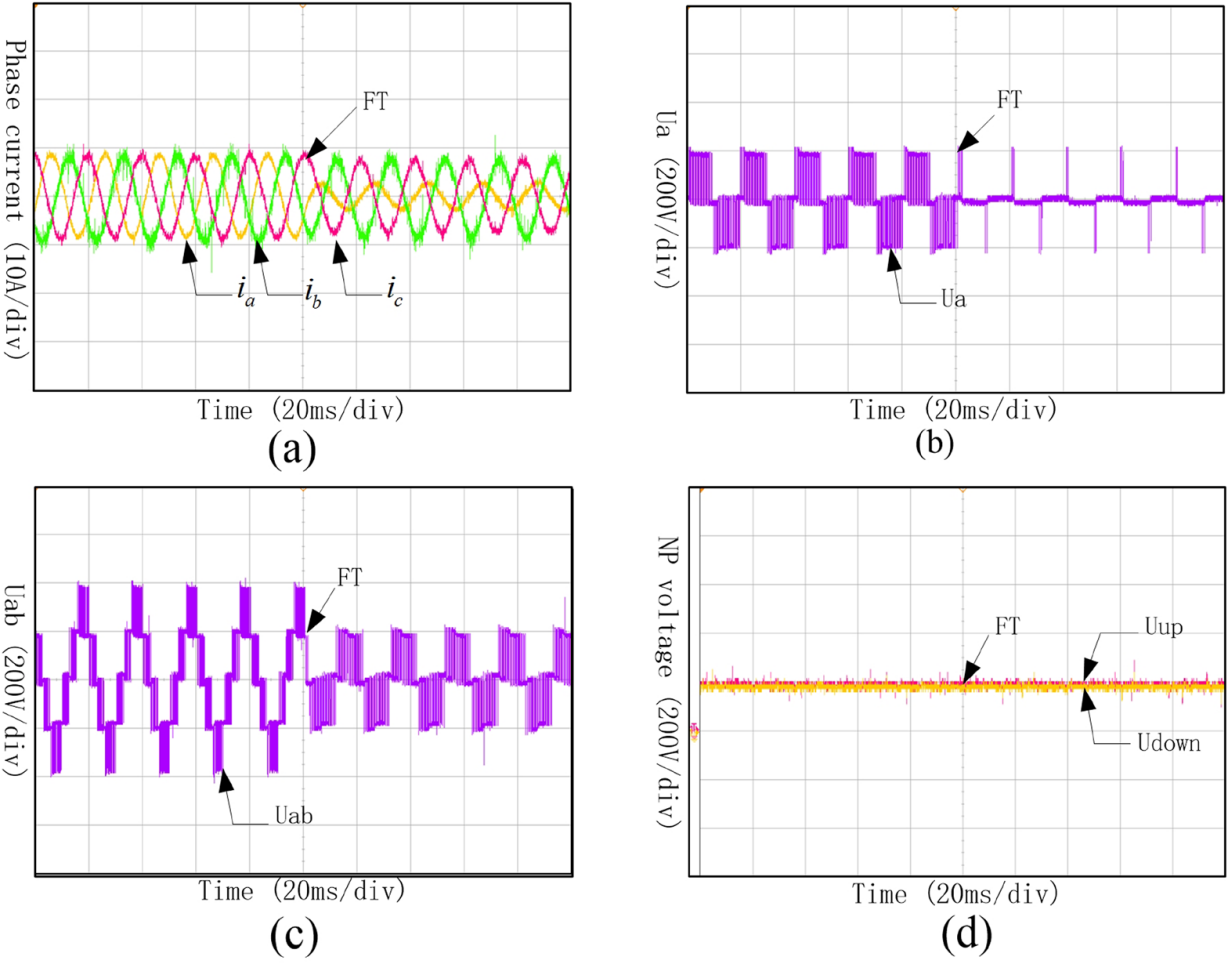
Waveforms of phase current, phase voltage, line voltage and neutral-point voltage under Sa1 and Sa4 open-circuit fault. (**a**) Three-phase currents under Sa1 and Sa4 open-circuit fault; (**b**) Phase voltage of phase A under Sa1 and Sa4 open-circuit fault; (**c**) Line voltage between phase A and B under Sa1 and Sa4 open-circuit fault; (**d**) Neutral-point voltage under Sa1 and Sa4 open-circuit fault.

It can be found from Fig. 13(a) that, when Sa1and Sa4 come into open-circuit fault at the same time, the three-phase current was no longer symmetric, and the current of phase A changed most obviously. It can be found from Fig. 13(b) and (c) that phase voltage of phase A and line voltage between phase A and B distorted because of the open circuit of Sa2. As shown in Fig. 13(d), the neutral-point voltage shifted a little, but it still could keep balance.

**Figure 14:**
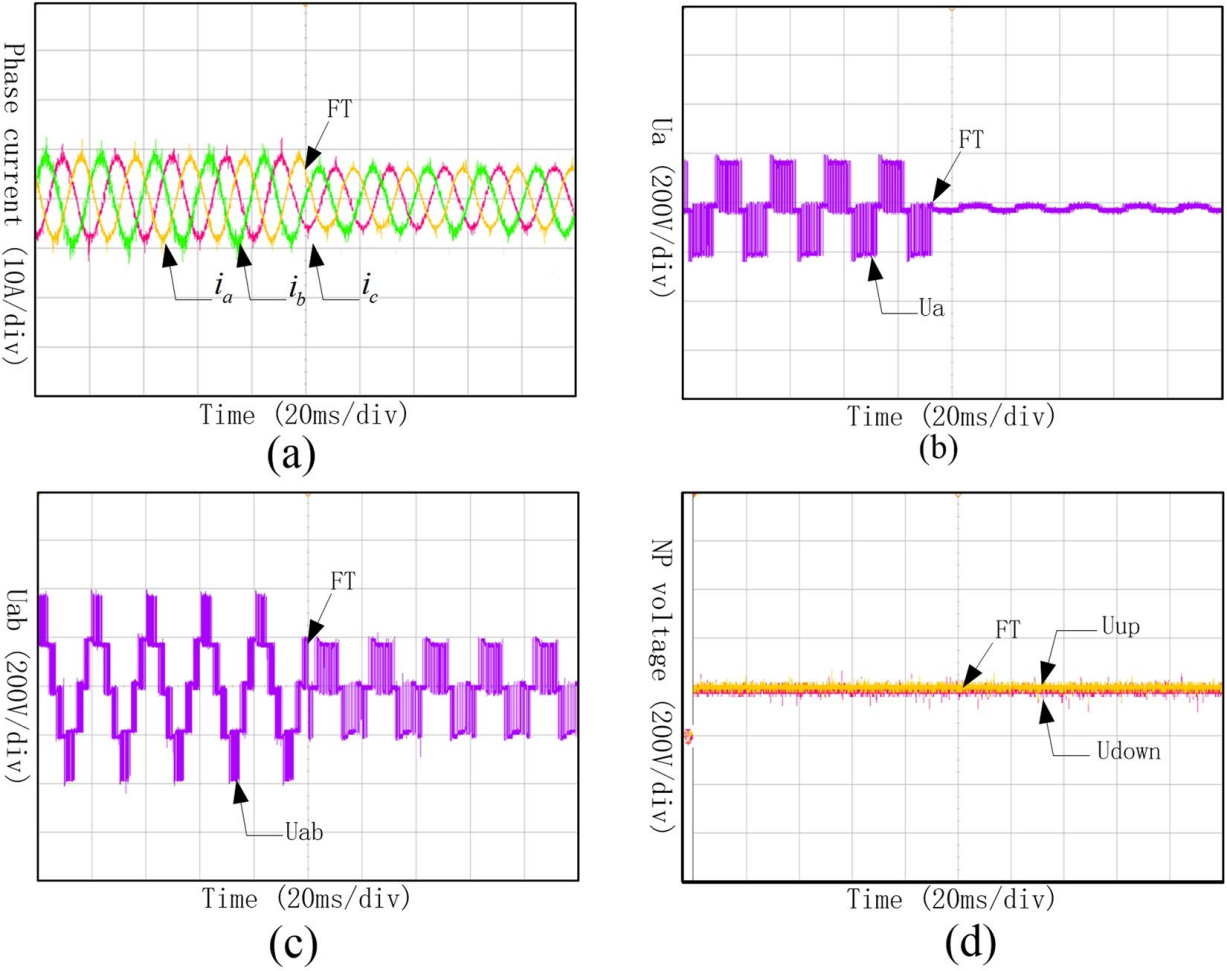
Waveforms of phase current, phase voltage, line voltage and neutral-point voltage under Sa1 and Sa4 open-circuit fault with fault-tolerant control. (**a**) Three-phase currents under Sa1 and Sa4 open-circuit fault with fault-tolerant control; (**b**) Phase voltage of phase A under Sa1 and Sa4 open-circuit fault with fault-tolerant control; (**c**) Line voltage between phase A and B under Sa1 and Sa4 open-circuit fault with fault-tolerant; (**d**) Neutral-point voltage under Sa1 and Sa4 open-circuit fault with fault-tolerant control.

It can be found from Fig. 14(a) that, when Sa1and Sa4 came into open-circuit fault at the same time and operated with fault-tolerant control, the amplitude of the three-phase current would decrease a little, but they are still symmetry. It can be found from Fig. 14(b) that phase voltage of phase A changed into 0 during the fault-tolerant control operation after the fault occurrence. As shown in Fig. 14(c), even though the line voltage between phase A and B decreased, it still can keep symmetry. Meanwhile, the neutral-point voltage which is shown in Fig. 14(d) can still keep balance.

**Figure 15:**
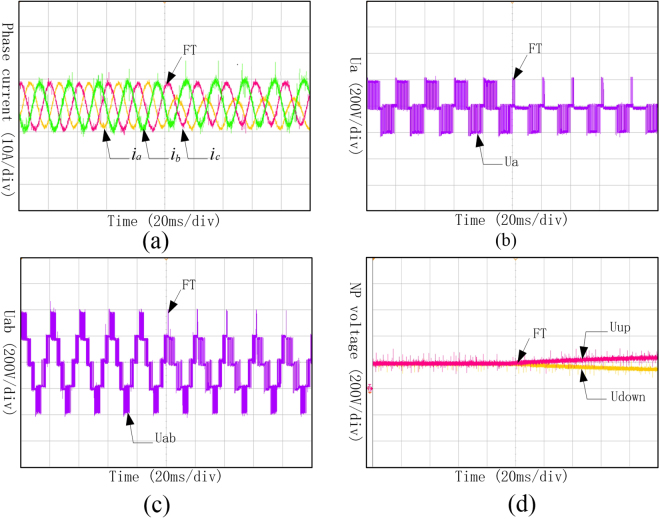
Waveforms of phase current, phase voltage, line voltage and neutral-point voltage under Sa1 and Sa5 open-circuit fault. (**a**) Three-phase currents under Sa1 and Sa5 open-circuit fault; (**b**) Phase voltage of phase A under Sa1 and Sa5 open-circuit fault; (**c**) Line voltage between phase A and B under Sa1 and Sa5 open-circuit fault; (**d**) Neutral-point voltage under Sa1 and Sa5 open-circuit fault.

It can be seen from Fig. 15 that the Three-phase currents under Sa1 and Sa5 open-circuit fault are no longer symmetry, and the current of phase A changed most significantly. Phase voltage of phase A and the line voltage between phase A and phase B distorted because of the open-circuit fault of Sa2. Meanwhile, the neutral-point voltage shifted under this condition, and lost the ability of keeping balance.

**Figure 16:**
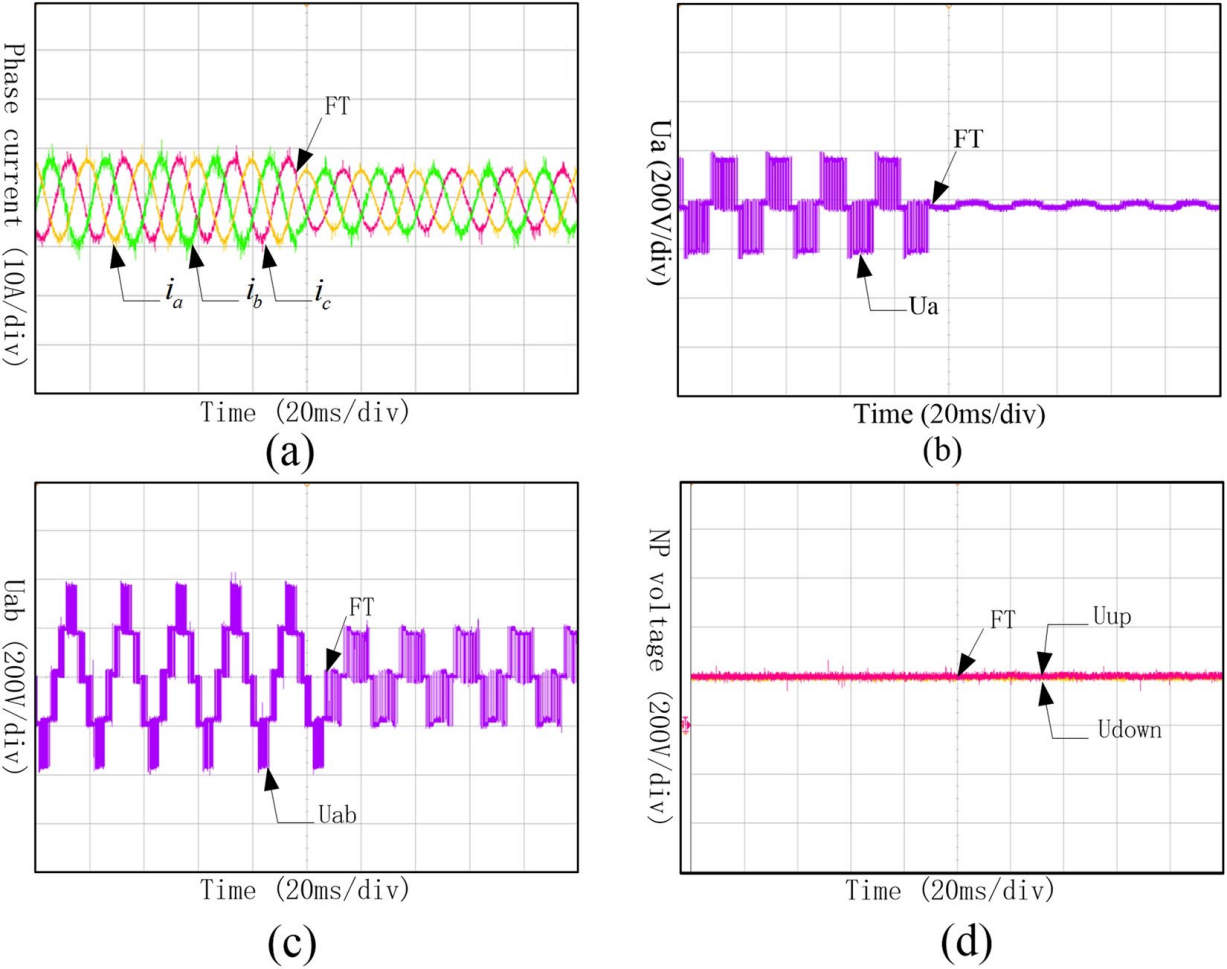
Waveforms of phase current, phase voltage, line voltage and neutral-point voltage under Sa1 and Sa5 open-circuit fault with fault-tolerant control. (**a**) Three-phase currents under Sa1 and Sa5 open-circuit fault with fault-tolerant control; (**b**) Phase voltage of phase A under Sa1 and Sa5 open-circuit fault with fault-tolerant control; (**c**) Line voltage between phase A and B under Sa1 and Sa5 open-circuit fault with fault-tolerant; (**d**) Neutral-point voltage under Sa1 and Sa5 open-circuit fault with fault-tolerant control.

It can be seen from Fig. 16(a) that even though the amplitudes of the three-phase currents have been decreased, they are still in symmetry. Under the fault-tolerant control, the phase voltage of phase A, shown in Fig. 16(b), changed into 0 after the fault occurrence. The amplitude of line voltage between phase A and B also decreased, which is shown in Fig. 16(c), and still can keep symmetry as three-phase currents did. Meanwhile, the neutral-point voltage which is shown in Fig. 16(d) can still keep balance.

**Figure 17:**
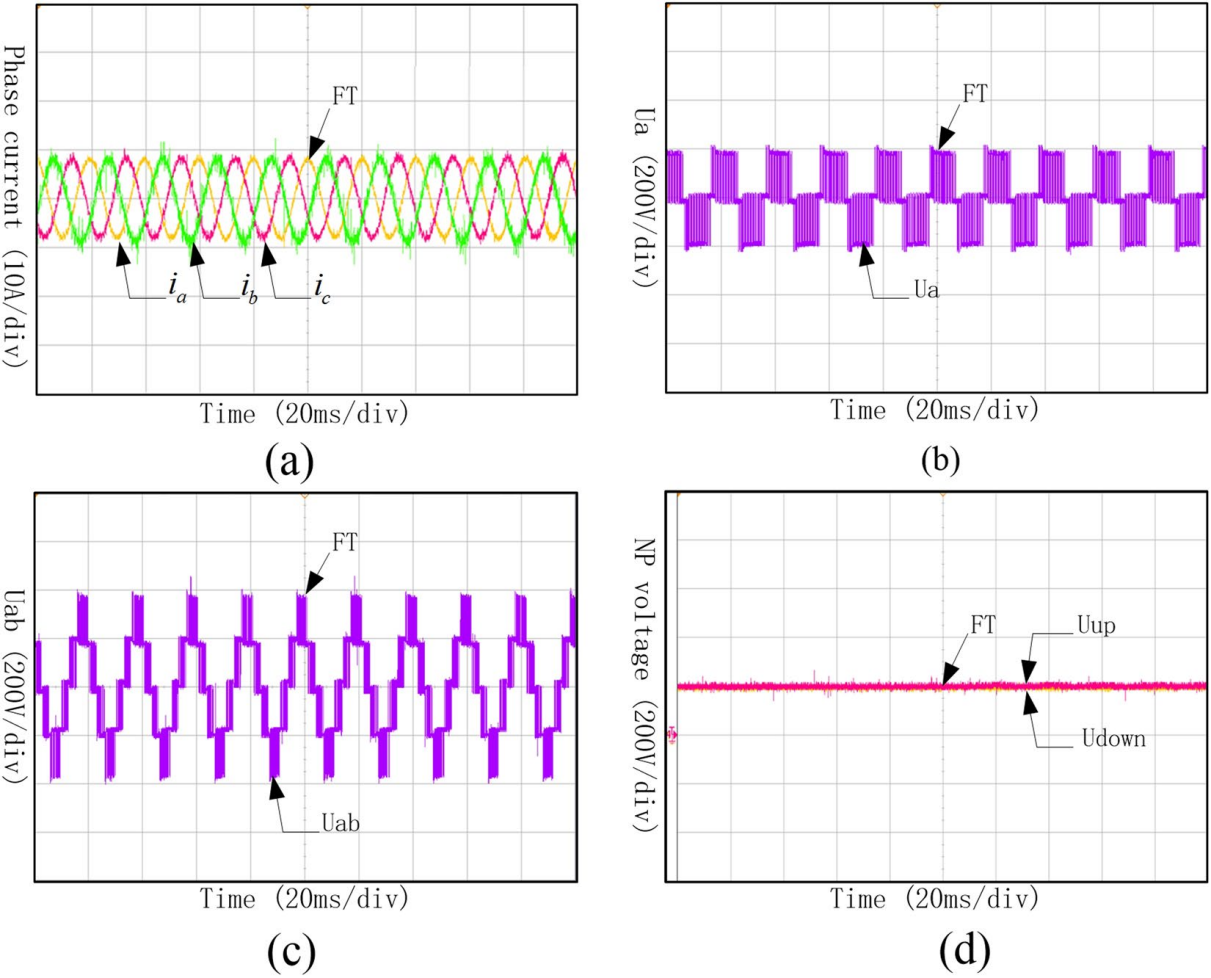
Waveforms of phase current, phase voltage, line voltage and neutral-point voltage under Sa5 and Sa6 open-circuit fault. (**a**) Three-phase currents under Sa5 and Sa6 open-circuit fault; (**b**) Phase voltage of phase A under Sa5 and Sa6 open-circuit fault; (**c**) Line voltage between phase A and B under Sa5 and Sa6 open-circuit fault; (**d**) Neutral-point voltage under Sa5 and Sa6 open-circuit fault.

The experiment results shown in Fig. 17 proved that the three-phase currents, phase voltage, line voltage and neutral-point voltage did not changed after the Sa5 and Sa6 open-circuit fault and can still output the same waveforms as the ones before fault occurrence. Therefore, it is unnecessary to apply the fault-tolerant control when Sa5 and Sa6 come into open-circuit fault, and the whole can maintain operating as normal.

These experiment results above are consistent with the simulation results. This order-reduction optimal control strategy can realize the fault-tolerant control to keep the system working in slowing down or output capacity reducing state to improve the reliability of the whole system.

## Conclusions

To improve the operation stability, this paper summarized all the possible states of multi-device open-circuit fault and proposed an order-reduction optimal control strategy to realize fault-tolerant control based on the topology and control requirement of ANPC three-level inverter and operation stability. This control strategy can solve the faults with different operation states, and can works in order-reduction state under specific open-circuit faults with specific combined devices, which sacrifices the control quality to obtain the stability priority control. Finally, the simulation and experiment proved the effectiveness of the proposed strategy. This paper may offer a practical method potentially to solve the multi-device open-circuit problem in ANPC three-level inverters.

### Data availability statement

The authors are responsible for ensuring that data will be available from the data owner post-publication, in the same manner as the authors obtained the data.
